# The Bidirectional Relationship between Gestational Diabetes and Depression in Pregnant Women: A Systematic Search and Review

**DOI:** 10.3390/healthcare11030404

**Published:** 2023-01-31

**Authors:** Samantha Fischer, María Morales-Suárez-Varela

**Affiliations:** 1 Bouvé College of Health Science, Northeastern University, 360 Huntington Avenue, Boston, MA 02115, USA; 2 Area of Preventative Medicine and Public Health, Department of Preventive Medicine and Public Health, Food Services, and Toxicology, School of Pharmacy, University of Valencia, Avenida Vincent Andres Estelles s/n, 46100 Valencia, Spain; 3 Biomedical Research Network Consortium on Epidemiology and Public Health (CIBERESP), Institute of Health Carlos III, Avenida Monforte de Lemos, 3-5, Pabellón 11, Planta 0, 28029 Madrid, Spain

**Keywords:** gestational diabetes, depression, history of depression, postpartum depression

## Abstract

This systematic search and review aims to understand the two-way relationship between gestational diabetes and depression. This study assesses gestational diabetes in relation to a history of depression, depression during pregnancy and postpartum depression. Searches were conducted on PubMed and Scopus. Studies were excluded due to being duplicates, not available, published before 2015 or did not include both gestational diabetes and depression. Of the 915 articles initially identified, 22 articles were included for review. Of the included studies, 18 were cohorts, 2 were case-controls, 1 was cross-sectional and 1 was a claims analysis. A meta-ethnography was conducted, and a bidirectional relationship was observed between a history of depression, depression during pregnancy, postpartum depression and gestational diabetes. Differing methodologies between studies were a limiting factor throughout this review. A two-way relationship between gestational diabetes and depression was observed; the diagnosis of gestational diabetes may lead to an increased risk of depression, both during the pregnancy and in the postpartum period, and a history of depression or symptoms of depression during pregnancy may lead to an increased risk of gestational diabetes.

## 1. Background

Gestational diabetes (GD) is one of the most common complications of pregnancy [1]. In 2021, it was estimated that 16.7% (21.1 million) of live births were affected by some form of hyperglycemia in pregnancy, and of these, 80.3% (16.9 million) were due to GD [1]. GD traditionally refers to an abnormal glucose tolerance with onset or first detection during pregnancy [2]. Global GD prevalence is estimated to be between 4 and 16%, depending on the ethnicity, geographic region and diagnostic criteria used [3]. According to a report published in 2020, the incidence of diabetes in women increased by 9% between 2011 and 2017 [4]. This increase may be due to physical inactivity, the obesity epidemic, and/or increasing maternal age [5,6]. The detection of GD is carried out between weeks 24 and 28 of gestation. In the United States, the 50 g Oral Glucose Challenge Test (OGCT) and the 100 g glucose Oral Glucose Tolerance Test (OGTT) are used to measure the body’s response to glucose and diagnose gestational diabetes [2]. GD is associated with several maternal complications in pregnancy, including pre-eclampsia, preterm labor, the need for induction of labor and cesarean delivery, as well as increased long-term risks of type 2 diabetes, metabolic syndrome, cardiovascular disease [7] and neonatal hypoglycemia [8]. Intrauterine exposure to GD raises the risk for complications in offspring as well, including stillbirth, macrosomia and birth trauma, and long-term risk of metabolic disease [9].

During pregnancy, 70% of women report symptoms of depression [10], and a recent systematic review estimated the antepartum depression prevalence rate at 16.4% [11]. Additionally, a rapid review and meta-analysis on the worldwide prevalence of depression among pregnant women during the COVID-19 pandemic estimated a pooled prevalence of 25.6% [12], while a similar study showed that the prevalence of depression was 30% [13]. The diagnosis of depression is usually based on clinical symptoms and signs established in the Diagnostic and Statistical Manual of Mental Disorders, Fifth Edition, DSM-V [14] or the International Classification of Disease, Eleventh Edition, ICD-11 [15]. The Edinburgh Postnatal Depression Scale (EPDS) [16] and Montgomery–Åsberg Depression Rating Scale (MADRS) [17] are typically used to identify symptoms of depression in pregnancy. Complications associated with depression during pregnancy include preterm birth and low birth weight, fetal growth restriction, hypertension and pre-eclampsia [10]. Postpartum depression (PPD) is a mood disorder that begins a week to a month after delivery [18]. Complications that can arise from PPD include impaired maternal–fetal bonding, shorter breastfeeding duration and/or long-term cognitive impairments in babies [19]. 

Qiu et al. (2021) found that symptoms of anxiety and depression can cause obesity and increased blood lipid and glucose levels [20]. Over the last three decades, clinicians have been seeing increasing numbers of patients with comorbid depression of various severity in diabetes [21], and the prevalence of metabolic syndrome was found higher in patients with psychiatric disorders [22], which may lead to type 2 diabetes. Additionally, depression is known to be more commonly diagnosed in women, with women being twice as likely as men to develop depression during their lifetime [23].

A possible association between depression, antidepressant use and metabolic disorders has been a controversial topic in the literature. The hypothesis suggests that depression and/or antidepressant use can influence blood glucose levels by inhibiting pancreatic insulin secretion, increasing cellular insulin resistance and/or indirectly affecting insulin secretion with weight gain [24]. Nevertheless, in other studies, there has been no observed increase in the risk of GD according to the depression status [25,26].

The main objective of this systematic search and review is to determine if there is a relationship between GD and the risk of depression before, during and after pregnancy.

## 2. Methods

### 2.1. Search for Evidence 

Two databases were used: PubMed and Scopus. These were selected because they are two of the most common and comprehensive databases for medical science research. The following search terms were used: “gestational diabetes” OR “pregnancy diabetes” AND “depression” OR “mental illness”. This search followed PRISMA guidelines. 

### 2.2. Screening and Inclusion Criteria 

**Figure 1:**
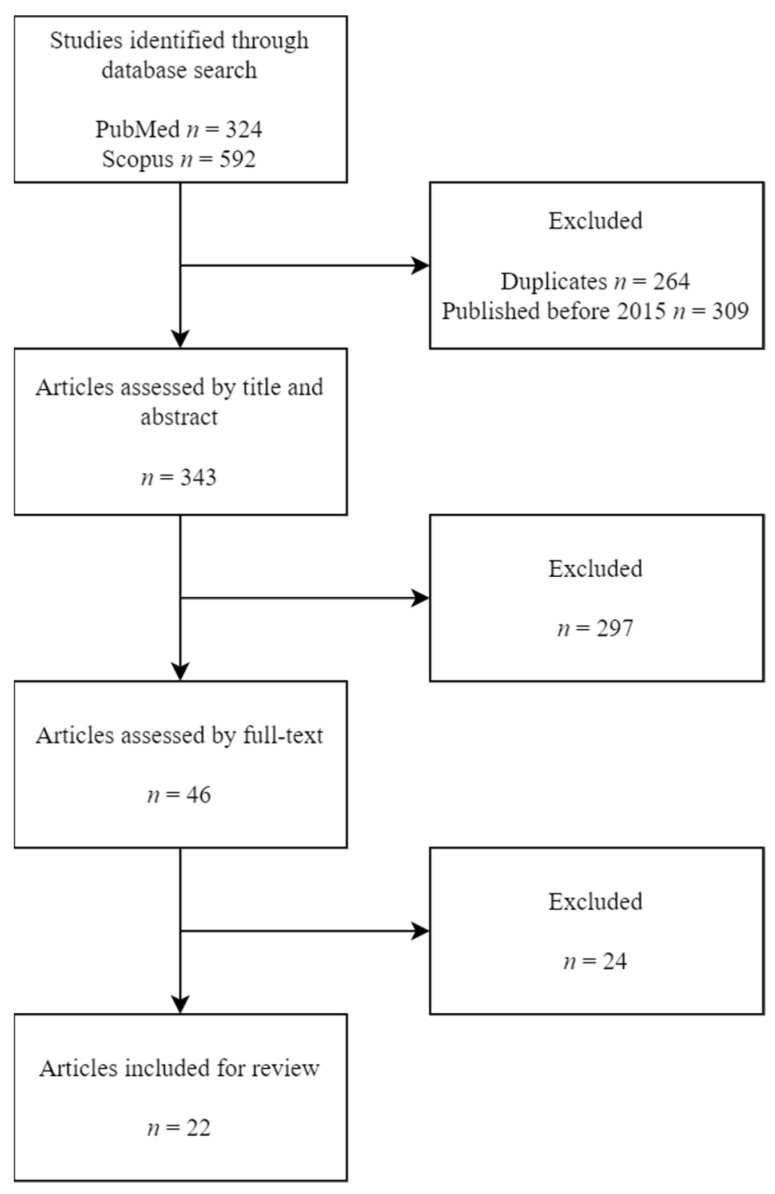
PRISMA flowchart.

Two authors conducted searches independently (SF and MMSV), using the same inclusion criteria. These criteria were full-text published meta-analyses, systematic reviews, observational studies (prospective and retrospective), cross-sectional or case-control articles; available in Spanish and/or English; published between 2015 and 2022; exposure/outcome of interest were GD and depression. Figure 1 shows the number of articles excluded at each stage of the eligibility assessment.

We initially identified 324 potentially eligible articles in PubMed and 592 in Scopus. Of these 916 initial candidate articles, 264 were excluded as duplicates, and 309 were excluded because their publishing date predated 2015. A total of 343 articles were assessed by their title and abstract, which led to the exclusion of 297 articles. The remaining 46 articles were retrieved for detailed full-text evaluation. Ultimately, 22 articles met all inclusion criteria and were included for review.

### 2.3. Data Collection and Synthesis 

The principal characteristics of the included studies are listed in Table 1, Table 2 and Table 3. Relevant data extracted include study design, year, country of origin, age of participants, measures of GD and depression and when GD and depression were tested for during the pregnancy. Outcome measures include incidence/prevalence of GD and depression, relative risk of developing depression during or after pregnancy, risk of developing GD in women with HD. A meta-ethnography was conducted, and an association between GD and the development of depression was observed [27,28,29,30,31,32,33], an association between GD and PPD was observed [28,29,34,35,36,37,38,39,40,41], and women with HD were observed to have a higher risk of developing GD [29,40,42,43,44,45]. Any heterogeneity in this review can be attributed to differing methodologies between studies: population size, variables measured, the diagnostic test used or control and experimental group sizes. Observational studies have an inherent risk of bias, which was considered in this review. 

## 3. Results

### 3.1. Study Selection

In the primary search, 915 studies were collected. After the initial screening of the title and abstract, the exclusion of duplicates and the elimination of those that were not available or those published before 2015, 45 studies remained. Finally, a total of 22 studies were included (Figure 1).

### 3.2. Study Characteristics

Of the 22 included studies, 18 were cohorts, 2 were case-controls, 1 was cross-sectional and 1 was a claims analysis. A total of three studies aimed to determine a relationship between HD and GD [42,43,44]; four studies aimed to determine a relationship between depression during pregnancy and GD [28,30,31,33]. Nine studies aimed to determine a relationship between GD and PPD [34,35,36,37,38,39,41,47]. Three studies included both depression during pregnancy and PPD in relation to GD [27,32,46]. One study included both HD and depression during pregnancy in relation to GD [45]. One study included HD and PPD in relation to GD [40]. One study included HD, depression during pregnancy and PPD in relation to GD [29] (Table 1, Table 2 and Table 3).

Sample sizes range from 70 [36] to 1,057,647 [31] (Table 1, Table 2 and Table 3). GD was determined in seven studies between 24–28 weeks of gestation [27,28,32,37,38,44,47]. In one study, it was determined in the third trimester (weeks 28–40) [46] and, in another study, in the first and second trimesters (weeks 8–13 and 16–22) [29]. In half of the articles, the time of measurement is not specified [30,31,34,35,39,42,43,45]. The timing of depression measurement is more variable. In five studies, it was not specified [30,33,41,42,43]; in five studies, depression was only determined only postpartum, 3 months after delivery [40], 6 months after delivery [45] or 1 year after delivery [34,39,46]; in eight studies, it was determined during pregnancy and after delivery [27,29,32,35,36,37,38,47]; in one study, only 2 years before pregnancy [44]; in another, it was determined from pregnancy to discharge [31]; and in another study, only at week 25 of gestation [28]. The variables taken into account vary by study but generally consider age, race/ethnicity, body mass index (BMI), parity, education and income. Only in three studies were the variables not specified [30,36,39]. 

### 3.3. Outcomes

The six studies on history of depression and risk of developing gestational diabetes showed that women who had depression before pregnancy had a higher risk of developing GD [29,40,42,43,44,45] (Table 1). In two studies, during pregnancy, the relationship between depressive symptoms and the risk of developing GD was sought, and an association was observed [29,43] (Table 2). It should be noted that in one study [43], the objective was to evaluate the effects of psychosocial factors on pregnancy, and one of the effects was GD; however, the other [29] was aimed at evaluating the relationship between depression and the risk of GD. The aim of 4 out of the 18 studies was to assess the relationship between GD and the risk of depression during pregnancy [28,32,33,46]. In all of them, a significant association was observed between GD and a higher risk of depression in pregnancy [27,28,30,31,32,33]. However, in one study [46], the association was not significant after adjusting for other clinical and demographic characteristics. A total of 11 studies sought to understand the risk of PPD or postpartum stress on GD [27,28,29,32,35,36,37,38,40,45,47], and 3 studies aimed to identify diabetes complications during pregnancy that lead to PPD, and one of them was GD [34,39,41] (Table 3). 

A meta-ethnography of the studies was conducted using the PICO method. The population studied was pregnant women. The main interest in studying this population was gestational diabetes and its relation to mental health issues. History of depression is a distinct characteristic that was included in the study, and its effects on the development of GD were measured. The variability is a limitation of this systematic search and review due to the different methodologies, variables and population sizes included in this study. Another limiting factor is the observational nature of many of the studies because it cannot prove causality, and these studies are more at risk for bias and confounding variables affecting the results. Additionally, most studies used measures of depression symptoms rather than empirical tests to diagnose depression (Table 1, Table 2 and Table 3). This may lead to information bias and rates of depression being underreported, especially in retrospective studies using clinical history. However, this review includes studies from all phases (before, during and after pregnancy) to analyze the relationship between gestational diabetes and depression, which increases the validity of bidirectional relationship theory. We can be certain about the validity of these results due to the fact that all studies examined both diabetes and depression despite the different methods for doing so. 

## 4. Discussion

This systematic search and review aims to understand the relationship between gestational diabetes and depression before, during and after pregnancy. It was observed that, despite conflicting data, there likely is a bidirectional association between gestational diabetes and depression in pregnancy.

### 4.1. Relationship between History of Depression and Risk of Gestational Diabetes

The results of this study suggest that HD before pregnancy is a risk factor for GD since a correlation was observed in all studies [29,42,43,44,45,49]. Likewise, a significant association was found between the increase in depressive symptoms during pregnancy and the risk of GD [29,30,31,32,43]. However, a systematic review [50] did not reach a consensus on the relationship between depression and GD due to the variability in the articles, the small sample of some articles and the determination of depression by depressive symptoms and not by diagnostic techniques. It should be noted that this particular study was one of the first systematic reviews, collecting articles from 1995 to 2015. After 2015, publications began to increase. The mechanisms of depression that put women at risk for gestational diabetes are uncertain. Biologically, depression-related immune dysfunction activates the hypothalamic–pituitary–adrenal axis and the sympathetic nervous system, leading to increased production of inflammatory cytokines and stress hormones. These can interact with pancreatic β cells to induce insulin resistance. Therefore, systematic increases in proinflammatory cytokines and adipokines associated with depression could increase the risk of GD [29,45,47,49]. In addition, depression is associated with many lifestyle choices, such as a sedentary lifestyle and lack of physical activity, which increase the risk of diabetes [49].

### 4.2. Relationship between Gestational Diabetes and Depression during Pregnancy and Postpartum

On the other hand, GD was also significantly associated with an increased risk of depression [27,28,30,31,32,51]. In one study [47], an association was observed when adjusting only for the variables of age, pre-eclampsia and preterm birth, but when clinical and socioeconomic factors were taken into account, it was the only one where there was no association. Furthermore, significant relationships between GD and PPD were found in most studies [29,34,35,36,37,38,39,49,52]. However, in other studies, this relationship was not significant [27,32,45,47]. The absence of association may be due to the small sample size since the prevalence of gestational diabetes was 0.6% in one study [27], and only 100 women with GD were used in another [47]. On the other hand, two studies [32,45] had a detection bias since the diagnosis of depression was only recorded if the person went to the health system. Despite the discrepancies that were found in the relationship between GD and the risk of PPD, there are possible mechanisms to support this association, but they are not clear. A possible explanation is the relationship between GD and stress leading to PPD [27,29,35,37,38,47,52]. There are physiological mechanisms, explaining GD as a risk factor for depression during pregnancy and PPD. The process of abnormal glucose metabolism could partially induce the dysregulation of the hypothalamic–pituitary–adrenal axis, leading to elevated cortisol levels, which in turn are involved in depression [27,35,37,38,52]. Furthermore, women with GD are more likely to experience an increased cytokine-mediated inflammatory response and increased adipokine concentrations, which are considered to be associated with depression [27,29,33,37]. Therefore, despite the contradictory data, it could be said that there is a relationship between GD and PPD since an association was observed in most studies. 

### 4.3. Implications 

More research is needed to fully understand the bidirectional relationship between GD and depression. However, during pregnancy, many hormones’ levels increase, including reproductive hormones such as progesterone and estradiol, as well as other hormones of other biological systems (thyroid-stimulating hormone, cortisol, cortitropin-releasing hormone or prolactin); these hormones return to normal levels after partum [53,54]. These drastic hormonal changes are counteracted by balancing measures and could have consequences for endocrine diseases, such as diabetes, and for mental health [55]. Some studies have found bidirectionality of endocrine disease and depression [56]. On the other hand, other factors may also be involved in these pathologies. To further elucidate the link between diabetes and depression mechanisms, future studies are necessary that use endocrine disease history and psychiatric conditions of the women before, during and after pregnancy. One weakness of this review was the variability between studies. One possible way to reduce the impact of variability is to create a universal and empirical way to diagnose depression. This would be used on all women, not just women with symptoms. Having consistent variables would vastly improve the reliability of these studies and other reviews. Additionally, many of the studies did not analyze different racial/ethnic groups separately. We think these results would be interesting to see, as we know that many public health issues have a differential impact on communities based on factors other than strictly healthcare. 

### 4.4. Limitations

This review had several limitations. There are significant variations in the methodology, design, depression and GD assessment tools, the cut-off points adopted in the depression scales (the EPDS was used in six studies with cut-off points at 9, 10 and 13), the timing of the GD and depression measurements, the study population, the sample size and adjusted variables that might underestimate or overestimate the pooled result. It is important to note that the primary objective of some of the studies was not relevant, and the results that were collected were often secondary findings. Additionally, the observational nature of these studies increases the likelihood of biased results. More research is needed to understand the relationship between GD and depression. 

## 5. Conclusions

The main objective of this work was to review the currently available scientific literature in order to determine if there is evidence to support a relationship between GD and depression. The results suggest the existence of an association between GD and the risk of depression in both the prenatal and postpartum periods. A positive association between depression and the risk of developing GD was also identified. This serves to highlight the apparent bidirectionality of the relationship between GD and depression. However, association is not the same as causality, and this cannot be currently established with the available data. Future studies should attempt to establish causal links between GD and depression and/or identify common underlying endocrine factors that could be involved in the etiology of both GD and depression. Currently, given the complexity of the etiology of both GD and depression in pregnant women, the available information is limited but a better understanding of the relationship between GD and depression is important for the prevention of both.

## Figures and Tables

**Table 1:** Outcomes of studies evaluating the impact of history of depression in women with gestational diabetes.

Author, Year Published	Study Design	Measure of Depression	Results
Beka et al., 2018 [44]	*n* = 373,674 (25.7% with HD) Retrospective Cohort	ICD-10 ICD-9	Women with depression early in pregnancy had a 3.21-fold increased risk of developing GD (95% CI: 1.00–10.28). A total of 3.8% developed GD.
Clark et al., 2019 [45]	*n* = 1439 ((1) 25% with HD, (2) 27% with GD) Retrospective Case-Control	ICD-9	Pregnant women with a history of mood or anxiety disorders are more likely to develop GD (OR (95% CI): 1.10 (1.06–1.14))
Dahlen et al., 2015 [43]	*n* = 3092 (10.7% with GD) Retrospective Cohort	EPDS ≥ 13	Women with a score greater than 13 on the EPDS scale were more likely to develop GD (adjusted OR (95% CI): 1.85 (1.14–3.03), *p* < 0.025).
Hinkle et al., 2016 [29]	*n* = 2802 (3.7% with GD) Prospective Cohort	EPDS ≥ 10	Women with depression early in pregnancy had a 3.21-fold increased risk of developing GD (95% CI: 1.00–10.28).
Minschart et al., 2021 [40]	*n* = 1843 (12.5% with GD) Prospective Cohort	CES-D Health Survey (SF-36)	Women who developed GDM (231; 12.5%) had significantly more often depressive symptoms than NGT (1612; 87.5%) women (21.3% (48) vs 15.1% (239), odds ratio (OR) 1.52, 95% confidence interval (CI) (1.08–2.16), *p* = 0.017).
Schoenaker et al., 2019 [42]	*n* = 11,556 (4.6% with GD) Prospective Cohort	CES-D	HD was correlated with insulin use in pregnancy (*p* = 0.012).

CES-D: Center for Epidemiologic Studies Depression Scale; CI: Confidence Interval; EPDS: Edinburgh Postnatal Depression Scale; GD: Gestational Diabetes; HD: History of depression; ICD: International Classification of Diseases; SF-36: 36-item Short Form.

**Table 2:** Outcomes of studies evaluating depression during pregnancy in women with gestational diabetes.

Author. Year Published	Study Design	Measure and Timing of Depression	Results
Hinkle et al., 2016 [29]	*n* = 2802 (3.7% with GD) Prospective Cohort	EPDS ≥ 10 Timing: 1st and 2nd trimester of gestation	Increased depressive symptoms in the second trimester were significantly associated with a risk of developing GD (*p* = 0.01).
Huang et al., 2015 [27]	*n* = 2112 (0.6% with GD) Prospective Cohort	EPDS ≥ 13 Timing:- 27.9 weeks gestation	The likelihood of antenatal depression was significantly higher in women with hyperglycemia (>140 mg/dL) (adjusted OR (95% CI):2.17 (1.21–3.88) *p* = 0.0066).
Jovanovič et al., 2015 [30]	*n* = 839,792 (6.5% with GD) Retrospective Claims Analysis	ICD-9 Timing: N/a	The relative risk (95% CI) of depression in women with GD versus women without GD was 1.17 (1.12–1.21).
Natasha et al., 2015 [28]	*n* = 748 (51% with GD) Prospective Case Control	MADRS Timing: week 25	The prevalence of depression in women with GD (25.92%) is higher than that of those without GD (10.38%). The association between depression and GD was significant (*p* <0.001). Women with GD had higher mean MADRS scores [8.33 ± 7.23] than those without DG [4.42 ± 5.89].
Pace et al., 2018 [32]	*n* = 58,400 Retrospective Cohort	ICD-10 ICD-9 Timing: week 24, delivery	Compared to women without GD, women with GD had almost twice the risk of developing depression (adjusted HR (95% CI): 1.82 (1.28–2.59)).
Tasnim et al., 2022 [33]	*n* = 105 (100% with GD) Cross-sectional	MADRS Timing: N/a	Mild to severe antenatal depression was present in 36.2% of the subjects (i.e., 14.3%, 19% and 2.9% for mild, moderate and severe depression, respectively).
Walmer et al., 2015 [46]	*n* = 18,888 (3.7% with GD) Prospective Cohort	ICD-9 Timing: N/a	GD is significantly associated with a risk of increased depression (OR adjusted^to^ (95% CI): 1.46 (1.16–1.83), *p* = 0.001); however, the association was not significant after adjusting for other characteristics^b^ (adjusted OR (95% CI): 1.29 (0.98–1.70), *p* = 0.064).
Whiteman et al., 2015 [31]	*n* = 1,057,647 (4.9% with GD) Prospective Cohort	ICD-9 Timing: postpartum hospital discharge	GD was significantly associated with an increased risk of depression (adjusted OR (95% CI): 1.44 (1.26–1.65)).

CI: Confidence Interval; EPDS: Edinburgh Postnatal Depression Scale; GD: Gestational Diabetes; HR: Hazards Ratio; ICD: International Classification of Diseases; MADRS: Montgomery–Åsberg Depression Rating Scale; N/a: Not available; OR: Odds ratio.

**Table 3:** Outcomes of studies evaluating postpartum depression in women with gestational diabetes.

Author. Year Published	Study Design	Measure and Timing of Depression	Results
Clark et al., 2019 [45]	*n* = 1439 ((1) 25% with HD, (2) 27% with GD) Retrospective Case-Control	ICD-9 DSM-IV Timing of determination: 6 months postpartum	No correlation was found between the diagnosis of GD and the risk of PPD (*p* = 0.808).
Hinkle et al., 2016 [29]	*n* = 2802 (3.7% with GD) Prospective Cohort	EPDS ≥ 13 Timing: 1st and 2nd trimester of pregnancy and 6 months postpartum	Women with DG had a 4-fold increased risk of developing PPD (95% CI 1.17–13.65).
Huang et al., 2015 [27]	*n* = 2112 (0.6% with GD) Prospective Cohort	EPDS ≥ 13 Timing: 27.9 weeks of pregnancy and 6 months postpartum	Hyperglycemia during pregnancy was not associated with a probability of PPD (adjusted OR (95% CI): 1.22 (0.63–2.36) *p* = 0.34).
Liu et al., 2021 [41]	*n* = 133,313 Cohort	EPDS Timing: 2–24 weeeks	Risk factors associated with postpartum depression: gestational diabetes mellitus (OR = 2.71, 95%CI 1.78–4.14, I^2^ = 0.0%).
Mak et al., 2019 [38]	*n* = 1499 (15.8% with GD) Prospective Cohort	EPDS ≥ 9–13 Timing: week 32–37 of pregnancy and 1,3 months postpartum	Scores on the EPDS scale were higher in women with GD at one month after delivery (*p* = 0.02) and at 3 months after delivery (*p* < 0.01).
Meltzer-Brody et al., 2017 [39]	*n* = 392,458 (% with GD N/a) Retrospective Cohort	ICD-10 Timing: up to 1 year postpartum	GD was associated with higher rates of postpartum stress (IRR (95% CI): 1.42 (1.03–1.97)).
Miller et al., 2016 [47]	*n* = 305 (32.8% with GD and 10.8% with diabetes pre-pregnancy) Prospective Cohort	PHQ-9 Timing: 3rd trimester and postpartum visit	No relationship was observed between GD and PPD (adjusted OR (CI 95): 0.97 (0.45–2.10)).
Minschart et al., 2021 [40]	*n* = 1843 (12.5% with GD) Prospective Cohort	CES-D Health Survey (SF-36) Timing: 3 months postpartum	Compared to GDM women without depressive symptoms, depressed GDM women attended less often the postpartum OGTT (68.7% (33) vs. 87.6% (155), *p* = 0.002), remained more often depressed (37.1% (13) vs. 12.4% (19), *p* < 0.001), and had lower SF-36 scores postpartum.
Pace et al., 2018 [32]	*n* = 58,400 (50% with GD) Retrospective Cohort	ICD-10, ICD-9 Timing: week 24, delivery, 1 year postpartum	The risk of being diagnosed with PPD in both women with and without GD was inconclusive (adjusted HR (95% CI): 1.05 (0.84–1.30)).
Rasmussen et al., 2022 [48]	*n* = 888,989 Prospective Cohort	ICD-8, ICD-10 Timing: 6 months postpartum	Women with an endocrine disease history had a 40% (risk ratio 1.42, 95% CI 1.24–1.62) higher risk of a PPD episode. The higher risk of PPD was evident for previous GD: risk ratio 1.5, 95% CI 1.09–2.06, and current GD: risk ratio 1.33, 95% CI 1.09–1.62).
Ruohomäki et al., 2018 [37]	*n* = 1066 (14.1% with GD) Prospective Cohort	EPDS ≥ 10 Timing: week 28, 44 of pregnancy and 8 weeks postpartum	Compared to women without GD (9.4%), women with GD had a higher prevalence of PPD (16%) (*p* value = 0.014). There was a significant association between GD and increased risk of PPD (just OR (95% CI):2.23 (1.23–4.05); *p* value 0.008).
Silverman et al., 2017 [34]	*n* = 707,701 (0.5% with GD) Prospective Cohort	ICD-10, ICD-9 Timing: 1 year postpartum	GD was strongly associated with an increased risk of PPD, regardless of their history of depression (RR (95% CI): 1.70 (1.36–2.13); *p* < 0.01).
Varela et al., 2017 [35]	*n* = 117 (14.5% with GD) Prospective Cohort	EPDS ≥ 13 Timing: 3rd trimester and 1 week postpartum	Women with GD were more likely to develop PPD (adjusted OR (CI95): 4.69 (1.97–20.64)).
Walmer et al., 2015 [46]	*n* = 18,888 (3.7% with GD) Prospective Cohort	ICD-9 Timing; 1 year postpartum	GD was significantly predictive of mental health disorders (including depression, anxiety and others) within 3 months of delivery (adjusted OR (95% CI):1.38 (1.04–1.85), *p* = 0.028).
Zwolińska-Kloc et al., 2017 [36]	*n* = 70 (50% with GD) Prospective Cohort	HADS, MINI Timing: 5–8 months gestation, 2, 6, 26 weeks postpartum	Women with GD are more likely to suffer a depressive episode in the first 6 months after childbirth (OR (95% CI):1.33 (0.56–3.19)).

CES-D: Center for Epidemiologic Studies Depression scale; CI: Confidence Interval; DSM-IV: Diagnostic and Statistical Manual of Mental Disorders, Fourth Edition; EPDS: Edinburgh Postnatal Depression Scale; GD: Gestational Diabetes; HADS: Hospital Anxiety and Depression Scale; HR: Hazards Ratio; ICD: International Classification of Disease; IRR: Incidence Rate Ratio; MADRS: Montgomery–Åsberg Depression Rating Scale; MINI: Mini International Neuropsychiatric Interview; OR: Odds ratio; PHQ: Patient Health Questionnaire; PPD: Postpartum depression; RR: Relative Ratio.

## Data Availability

Not applicable.
